# Revealing structural evolution occurring from photo-initiated polymer network formation

**DOI:** 10.1038/s42004-020-0335-9

**Published:** 2020-07-10

**Authors:** C. J. Brett, S. Montani, M. Schwartzkopf, R. A. T. M. van Benthem, J. F. G. A. Jansen, G. Griffini, S. V. Roth, M. K. G. Johansson

**Affiliations:** 1 KTH Royal Institute of Technology, Department of Engineering Mechanics, Teknikringen 8, 10044 Stockholm, Sweden; 2 KTH Royal Institute of Technology, Wallenberg Wood Science Center, Teknikringen 36, 10044 Stockholm, Sweden; 3 Deutsches Elektronen-Synchrotron, Notkestrasse 85, 22607 Hamburg, Germany; 4 KTH Royal Institute of Technology, Fiber and Polymer Technology, Teknikringen 56, 10044 Stockholm, Sweden; 5 Politecnico di Milano, Department of Chemistry, Materials and Chemical Engineering “Giulio Natta”, Piazza Leonardo da Vinci 32, 20133 Milano, Italy; 6 Eindhoven University of Technology, Laboratory of Physical Chemistry (SPC), Groene Loper 5, 5600 MB Eindhoven, the Netherlands; 7 DSM Material Science Center, Urmonderbaan 22, 6167 RD Geleen, the Netherlands

**Keywords:** Polymer characterization, Photochemistry

## Abstract

Photopolymerization is a key enabling technology offering spatial and temporal control to allow for future functional materials to be made to meet societal needs. However, gaining access to robust experimental techniques to describe the evolution of nanoscale morphology in photo-initiated polymeric systems has proven so far to be a challenging task. Here, we show that these physical transformations can be monitored and quantified at the nanoscale in situ and in real-time. It is demonstrated that the initial structural features of the liquid precursors significantly affect the final morphology and the physical properties of the resulting solid via the occurrence of local heterogeneities in the molecular mobility during the curing transformation. We have made visible how local physical arrestings in the liquid, associated with both cross-linking and vitrification, determine the length scale of the local heterogeneities forming upon curing, found to be in the 10–200 nm range.

## Abstract (web summary)

Acomplete account of the structural evolution occurring during photopolymerisation is lacking. Here the physical changes occurring on the nanometer scale during photopolymerisation of acrylates are followed over time by FTIR, X-ray reflectometry, AFM, and GISAXS, offering insight into the mechanism by which initial composition influences the final morphology.

## Introduction

Photopolymerization is a widely used technique for the fabrication of materials with unprecedented application areas ranging from surface coatings to bone glues^1,2^. From a molecular point of view, the transition from liquid monomers to a cross-linked solid can be currently described based on a well-established understanding of the photo-chemistry as well as of the kinetics^3^ of the polymerization reaction. The simultaneous physical transformation occurring to the material during the photo-initiated process has been elucidated at the microscopic scale^4,5^, while in the nanoscale domain the description has been limited to hypothetical approaches and theoretical simulations^6,7^. Photopolymerization techniques have proven their capabilities in achieving materials of tailored optical, chemical and mechanical properties while providing access to advanced morphing structures where the three-dimensional Origami-like architecture of the solid part can be dynamically actuated with high lateral resolution during polymerization^8–12^. The photopolymerization of single-crystal two-dimensional polymers yielding molecular thin sheets has been described by means of X-ray diffraction^13^. However, a thorough understanding of the film formation process is however still lacking^13^. Recently GISAXS has been used to monitor film formation processes for physically drying dispersed systems on a larger size range and time scale^14,15^.

The present study demonstrates that both chemical and physical transformations during photopolymerization can be monitored in situ and real-time by combining traditional techniques such as real-time Fourier-transform infrared spectroscopy (FTIR) with advanced synchrotron characterization techniques such as grazing-incidence small-angle X-ray scattering (GISAXS). We clearly show that a correlation exists between the initial nanoscale structures present in the monomeric precursors and the final nanosized domains in the resulting solid polymer. Our results further demonstrate that the local morphology in photocurable thermoset resins can be tailored on the basis of the choice of the liquid precursors and their associated glass transitions, thereby enabling a refined control over the macroscopic properties of photocured materials through a predictive design at the nanoscale^16,17^.

## Results and discussion

### Photo-initiated conversion

**Fig. 1:**
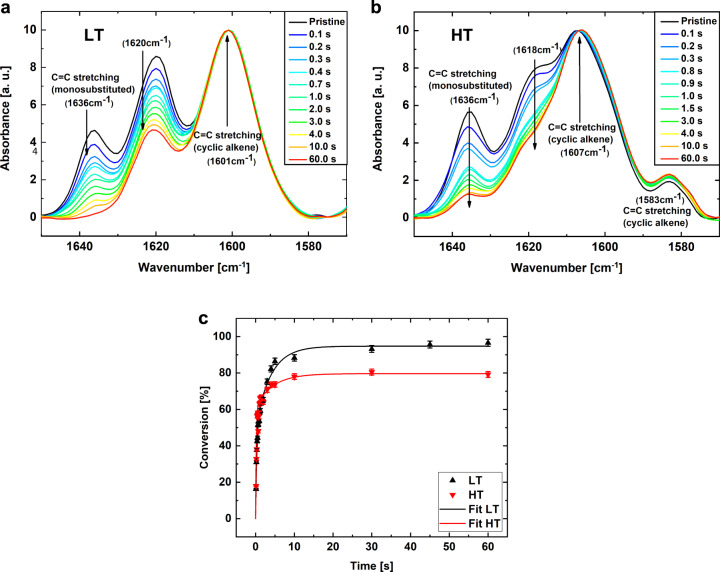
Most pronounced changes in chemical bonds during the photo-initiated polymerization process. **a** shows the absorbance spectra for LT sample, where it is clearly observed that the C=C monosubstituted stretching peak at (1636 ± 1) cm^−1^ vanishes completely during UV-induced polymerization, allowing nearly 100% conversion. **b** shows the absorbance spectra for the HT samples, where the C=C monosubstituted stretching peak at (1636 ± 1) cm^−1^ is found to only decrease in size but does not vanish completely. The graphs show the pristine sample, and polymerized samples after 0.1 s, 0.2 s, 0.3 s, 0.8 s, 1.0, 1.5 s, 2.0 s, 3.0 s, 4.0 s, 10.0 s, and 60.0 s of UV exposure. **c** Conversion of the two polymer resins measured using FTIR, LT in black and HT in red. The resins are polymerized step-wise and full FTIR spectra are recorded for each step, from these spectra the relative intensity of the C=C monosubstituted stretching peak at (1636 ± 1) cm^−1^ is used to calculate the conversion. The data is then fitted using an exponential function represented by the solid lines. The fitting parameters are presented in Supplementary Table 3.

Two different well-defined model photopolymer formulations (denoted LT (low *T*_*g*_) and HT (high *T*_*g*_)) were chosen to visualize the differences in performance all the way from the initial state until the final cross-linked solid state. LT, with an ultimate glass transition temperature *T*_*g*_ well below ambient temperature, and HT, with a *T*_*g*_ above it, allowed for a comparison between a system polymerizing completely in the non-vitrified state (LT) and a system undergoing vitrification during polymerization (see Supplementary Fig. 1f). Both LT and HT are acrylate functional resin formulations reacting via a chain-growth mechanism to form a cross-linked polymer network (Supplementary Table 1, Supplementary Fig. 1). It is well established that acrylate-based networks are prone to form rather inhomogeneous structures with density fluctuations within the material^18,19^. Density fluctuations in chain-growth polymerization effected network formation, such as particularly photopolymerizing acrylates, are known to nucleate by nano-size phase separation of fast-growing network fragments (of high molecular weight) caused by their poor solubility in the network precursors (unreacted “monomers”)^4,20,21^. These growing network fragments still contain free radical chain ends and continue to grow in molecular weight by reaction with dissolved monomers in their gel domains, see Supplementary Fig. 2. These initial gel domains grow by a reaction-diffusion mechanism, as continuous diffusion of unreacted monomers, driven by their reactive depletion, takes place into these reactive domains. If a fraction of residual acrylate groups remains unreacted because vitrification has arrested the conversion, local differences in reactive shrinkage result in local density differences^22^. Alternatively, the reaction-diffusion mechanism leads to a high degree of chain stretching, i.e. limited rotational freedom, of the initial network fragments as they are continuously swollen by the influx of new monomers that polymerize as a “multiple network” inside their gel domain^23^. In our case, both formulations polymerized very rapidly as monitored with real-time FTIR (Fig. 1, Supplementary Fig. 1g), with LT reacting to full double bond conversion and HT to 80% conversion. The residual non-reacted double bonds in the HT film are due to a combination of vitrification and restricted topological mobility effects in this more densely cross-linked network.

### In plane morphology

**Fig. 2:**
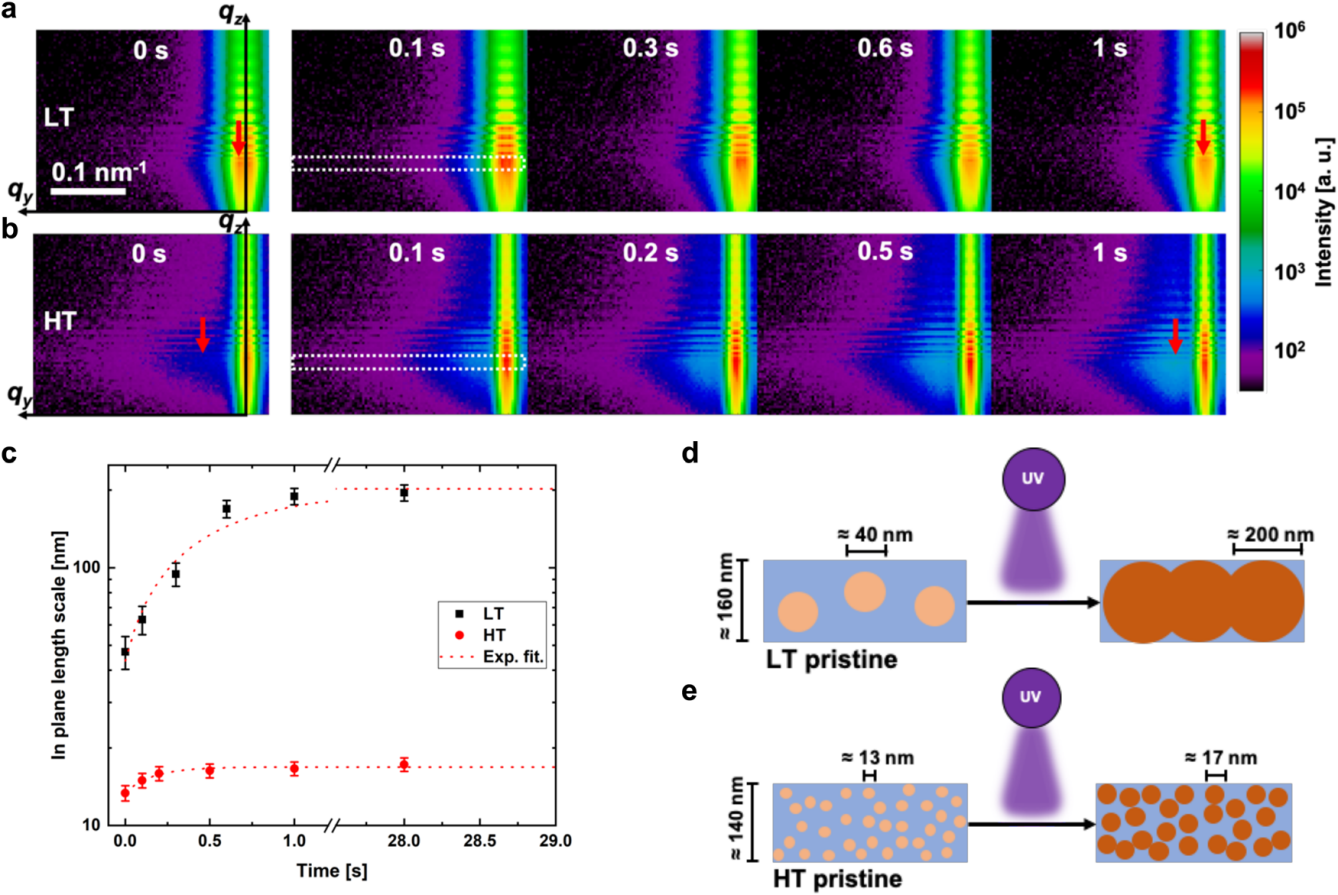
Grazing-incidence small-angle X-ray scattering patterns and the extracted domain sizes with increasing polymerization time as well as a schematic representation of the polymerization process. **a**, **b** Show the summed scattering patterns for each individual polymerization time, LT in **a** (pristine = 0 s, 0.1 s, 0.3 s, 0.6 s, and 1 s) and HT in **b** (0 s, 0.1 s, 0.2 s, 0.5 s, 1 s). The vertical red arrow marks the feature peak position in the pristine and the 1 s polymerized sample. The white dashed box in 0.1 s shows the Yoneda region which is integrated in q_y_-direction for fitting. **c** shows the extracted in plane length scale for LT (black) and HT (red) with an exponential fit which follows the same trend as the conversion in the FTIR spectra, see also Supplementary Fig. 1. The error bars are fit errors. **d**, **e** show schematically the network formation inside the film during the photo-initiated polymerization for LT and HT, respectively. On the left the pristine film with small domains, which increase in size and merge till the film is fully polymerized. We propose that the length scale growth is restricted due to the confined thickness of the film of around 140–160 nm. We interpret the peak shift in the GISAXS patterns with in plane length scale growth which is shown with the circles with increasing size when irradiated with UV light. The blue matrix is the unpolymerized surroundings, which is also correlated with the not full conversion followed by FTIR.

**Fig. 3:**
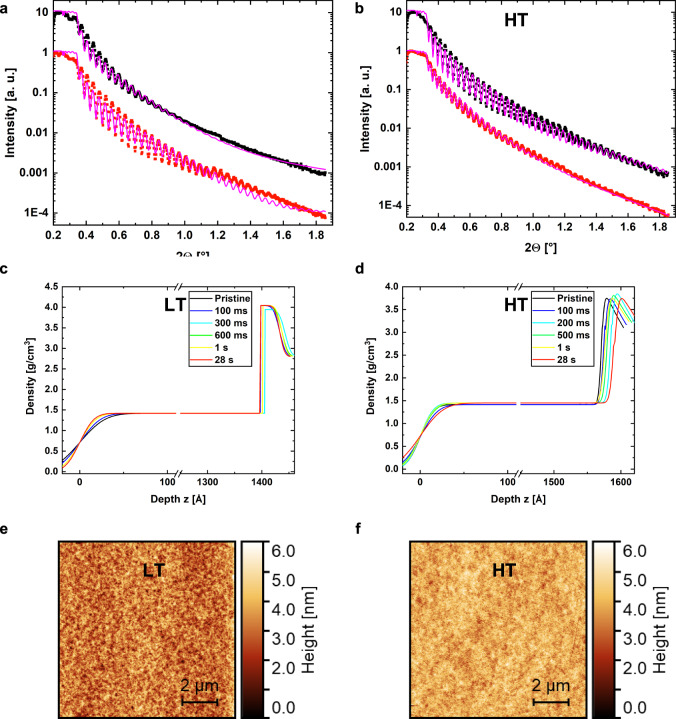
Characterization by X-ray reflectivity and atomic force microscopy. The X-ray reflectivity (XRR) data including a fit of the pristine and fully photopolymerized (28 s) film are shown for LT in **a** and HT in **b**. Density curves calculated from XRR curves for each polymerization time step of (0 s, 0.1 s, 0.3 s, 0.6 s, 1 s, 28 s) for LT in **c** and (0 s, 0.1 s, 0.2 s, 0.5 s, 1 s, 28 s) for HT in **d**. In **e** and **f** are the corresponding AFM topographical maps of the fully polymerized (28 s) films for LT and HT respectively. The surface rms-roughness of the fully polymerized sample is σ_LT_ = (0.74 ± 0.07) nm and σ_HT_ = (0.55 ± 0.07) nm, as extracted from the AFM images. The thicknesses of the thin films were fitted by the distances in the Kiessig fringes of their XRR profiles: d_LT, 28s_ = (143.2 ± 1.5) nm, d_HT, 28s_ = (160.1 ± 1.9) nm, which are confirmed by measuring the depth of a scratch in the fully polymerized film with AFM. The XRR patterns are divided in two regimes to study the evolution in peak-to-valley distance and therefore the correlated roughness at the air/polymer and polymer/substrate interface, see Supplementary Fig. 6.

Figure 2a shows the temporal evolution of the nanoscale morphology as measured by GISAXS for different polymerization times (upper row LT, lower row HT). Strong Kiessig fringes can be observed in both cases. They indicate that smooth homogeneous films with a surface roughness of below 1 nm can be achieved, together with a thickness after 28 s of photopolymerization of *d*_*LT, 28s*_ = (143.2 ± 1.5) nm and *d*_*HT, 28s*_ = (160.1 ± 1.9) nm for LT and HT respectively. During intermittent photopolymerization steps only minor changes of the Kiessig fringes are observed. This is indicative that the film retains a structural integrity without any macroscopic defect or delamination (see also Fig. 3). In horizontal direction, there are clear side maxima observed for LT, while the side maxima for HT are less pronounced and observed at higher *q*_*y*_. The maxima were previously associated in polymer blends to reoccurring domains within thin films^24^. With intermittent photopolymerization, the side maxima for LT shift to lower *q*_*y*_ and diminish clearly after 0.6 s. The same trend is observed for the position of the side maxima in HT, but increasing in intensity with increased polymerization time. After 1 s of UV exposure the side maxima are still clearly detectable. This means that heterogeneities are present in both LT and HT already in the pre-cured state and that these nanoscale features grow in size as the polymerization proceeds. From the difference in *q*_*y*_ positions of these side maxima, it can be concluded that the prominent in plane length scale of these heterogeneities differ significantly in the two polymeric systems considered, being much smaller for HT. We propose that this evidence is related to the difference in glass transition temperature between LT and HT, i.e. to differences in segmental mobilities^25^. The quantitative analysis of the film morphology is shown in Fig. 2b. Here, the in plane length scales of such heterogeneities were deduced (as outlined in the SI) and were found to grow in LT from (47.2 ± 4.3) nm to (189.2 ± 12.4) nm during 1 s of UV exposure. For longer photopolymerization times, they stay constant. The growth rate can be found in Supplementary Table 3. The thin films show correlated roughness with the substrate beneath already after spin-coating. Correlated roughness in this context denotes the average in-plane roughness which translates (replicates) the roughness of the underlaying substrates on the surface to the polymer film above^26^. The polymerization does not change the correlation to the substrate (see Fig. 2a, Supplementary Fig. 3). The minimum correlation length for LT is $R_{corr,LT} =$ (52.3 ± 4.1) nm.

The growth behavior for the heterogeneities in HT is different, with their characteristic in plane length scale changing from (13.4 ± 0.1) nm to (17.2 ± 0.2) nm during photopolymerization. The growth of these heterogeneities is diminished already after 0.5 s. The minimum correlation length within the HT resin is also unaffected by the polymerization time, yet highly reduced to $R_{corr,HT} =$ (28.6 ± 1.2) nm. The correlation length difference is ≈ 20 nm and as the differences in growth kinetic between LT and HT are related to a different molecular cooperative segmental mobility in the two systems, as also seen from the different *T*_*g*_ (LT = −17.3 °C and HT = 40.5 °C)^27^. Fig. 2c, d picture the different characteristic nanodomain dimensions determined for both systems. For LT, the in plane length scale of the heterogeneities after photopolymerization is in the same range as the film thickness, while HT shows heterogeneity domains well below the film thickness. In this latter case, we propose that the heterogeneities are homogenously distributed throughout the film thickness as they do not grow or alter anymore during further UV-treatment. Comparing these results with the FTIR data, it is clearly seen that the evolution of the morphology mainly occurs at the early stage of the polymerization while later stages of chemical transformations result in fewer changes of the nanostructures. This emphasizes the importance of the resin formulation for the final morphology in the nanometer region.

### Interface evolution

To probe the internal interfaces, roughnesses and homogeneities throughout the thickness of the thin films, we employed X-ray reflectometry (XRR) at different polymerization times for LT and HT, Fig. 3a, b, Supplementary Fig. 6, Supplementary Note 1. In addition, atomic force microscopy (AFM) was used to probe their surface topography, yielding root-mean square roughnesses of σ_LT_ = (0.74 ± 0.07) nm and σ_HT_ = (0.55 ± 0.05) nm for the fully polymerized LT and HT samples, respectively. The density profiles along the thickness of the films present a clear trend, see Supplementary Fig. 6c. As inferred from XRR measurements on HT at the air-resin interface (a free surface), the film roughness decreases strongly from (2.3  ± 0.2) nm to (1.5 ± 0.1) nm within 300 ms of UV exposure. It is constant until 1 s and finally decreases to (1.3 ± 0.1) nm for a polymerization time of 28 s. This is very well seen in Supplementary Fig. 6a, where the peak-to-valley ratio of the fringes increases strongly in the low *2θ* region from 0.4° to 1.0°. For HT the roughness at both interfaces (air-resin and resin-substrate) show the same trend, being considerably smaller at the resin-substrate interface due to the very smooth silicon wafer substrate. For LT, the roughness at the resin-substrate and at the air-resin interface increases as the polymerization proceeds, the latter being constant at around (1.3 ± 0.2) nm during the first second of UV exposure and then increasing to (2.1 ± 0.1) nm after *t* = 28 s. Thus, the two systems show opposite trends. The corresponding Guinier radii of the uncured formulations, as measured by SAXS (Supplementary Fig. 3a) yields (1.84 ± 0.10) nm for LT and (1.46 ± 0.08) nm for HT. We propose that the final surface roughness at a fully cured state is determined by the average initial size of the Guinier radii. A larger size correlated to a lower cross-linking density (amount of covalent bonds formed per unit volume) will lead to a larger final surface roughness. However, the trend observed on the evolution of surface roughness during polymerization differs, due to differences in initial average composition of the two polymer systems, where HT exhibits initially a low surface roughness (small average in plane length scale) and LT a correspondingly lower surface smoothness (higher average in plane length scale).

It should be noted that AFM measures the local interface fluctuations while XRR probes density fluctuations in the interphase over a larger averaged area. This combined AFM and scattering study clearly shows that the two systems differ both with respect to the internal morphology as well as to the size of nanoscale heterogeneities developing at the resin-air interface. Further differences are observed in the heterogeneity distribution and gradient along the film thickness from the interface to the bulk of the film. In particular, LT exhibits a larger surface roughness as determined by AFM, while it shows a lower density gradient/roughness as determined by XRR. Here the density gradient refers to the change in density along the surface normal.

In summary, we here demonstrate for that it is possible to correlate chemical transformations taking place during photopolymerization of acrylate-based resins with their morphological evolution at the nanoscale using a combination of traditional techniques (FTIR, AFM) and advanced synchrotron-based scattering methods, in situ and real-time. We show the existing correlations between the chemical formulation of the starting monomer precursors, their initial nano-morphology in the early phase of the polymerization process and the final nano-morphology of the cured systems, in relation to the macroscopic properties of the resulting materials. The results obtained here provide a deeper insight into the photo-induced formation of structural polymer networks, thereby allowing the tailored engineering of high-performance materials in fields as diverse as advanced electronics and 3D printing.

## Methods

### Reagents

The two used resin formulations are based mainly on polyether urethane oligomer. The theoretical average molecular weight is M_n_ = 2850 g/mol. The low T_g_ resin (LT) consists of 71.4 wt% of the oligomer and 28.6 wt% of 2-ethylhexyl acrylate. The high T_g_ resin (HT) is composed of 40.8 wt% of the oligomer, 18.4 wt% of 2-ethylhexyl acrylate and 40.8 wt% of bisphenol A diglycidyl ether diacrylate. Bisphenol A diglycidyl ether diacrylate is added to alter the *Tg* of the resin. Both resins are thoroughly mixed with a magnetic stirrer for 8 h and 500 rpm with 0.25 wt% of Irgacure® 651 serving as photoinitiator. The formulation stability is checked by monitoring the weight loss in time at room temperature, after depositing 100 mg of resin on a glass substrate (AB54-S, Mettler Toledo). The weight loss is about (0.9 ± 0.1)%/h and (0.5 ± 0.1)%/h for LT and HT, respectively. For spin coating the formulations are mixed 1:9 w/w with n-butyl acetate (≥99%, Carl Roth GmbH) and stirred for another 6 h at 300 rpm. Polished boron-doped silicon wafers (Si-Mat GmbH) were cut in (12 × 12) mm^2^ pieces. The wafers were cleaned in an acetone (≥99%, VWR Chemicals) ultrasonic bath for 15 min. Subsequently, the wafers were rinsed with acetone, 2-propanol, ethanol (≥99%, ≥98%, 96% respectively, all VWR Chemicals) and ultra-pure water (18.2 MΩ cm^−1^). The wafers were kept in ultra-pure water till used. Before spin-coating, the wafers were dried using a nitrogen flow.

### Preparation of films

Both diluted resins were spin-coated on the aforementioned cleaned silicon wafers. 0.2 ml of the compositions were then dispensed on the wafer using a pipette. The spin-coating process (Chemat Technology INC, KW-4A) was performed at 6000 rpm for 60 s. The obtained thicknesses were confirmed by atomic force microscopy and X-ray reflectivity measurements. For some characterization techniques thicker films were needed (e.g. dynamic mechanical analysis), while for X-ray techniques we chose thin films with a few hundred nanometer thickness. During the spin-coating process the light in the room was kept at a minimum to prevent polymerization before starting to analyze.

### UV-photopolymerization

The obtained films were irradiated subsequently under a UV-LED (365 nm) (FireJet FJ800, Phoseon Technology) using a power density of 11.4 mW/cm^2^. For real-time FTIR, an Hg/Xe light source equipped with a 365 nm (10 nm FWHM, Edmund Optics) bandpass filter was used to have the same spectrum as the UV-LED. The light intensity was checked using a Hamamatsu (C6080-03) light power-meter. Every photo polymerization was performed in a special nitrogen-purged chamber. This chamber allowed in situ X-ray measurements as well as optical checks using Vis-spectroscopy and microscopy.

### Fourier-transform infrared spectroscopy

Real-time FTIR was conducted to confirm the transformation from the resins to a fully cured film. Therefore, we spin-coated the films and irradiated (11.4 mW/cm^2^) the films step wise with different light doses. Due to the fact that the curing mechanism is of radical type, the process stops when the UV light is turned off. This fact makes it easy to monitor the conversion in time. All FTIR spectra are normalized to the C=C cyclic alkene stretching at (1601 ± 1) cm^−1^ and (1607 ± 1) cm^−1^ for LT and HT, respectively. This could be done as the same sample was measured for all UV-curing steps. The conversion is calculated using several FTIR curves and observing the decrease in the monosubstituted C=C stretching (1636 ± 1) cm^−1^, see Fig. 1. The data are then converted in the form of a conversion (%) versus exposure time plot and fitted using an exponential function, see Supplementary Fig. 1.

### X-ray scattering

The grazing incidence and transmission small-angle X-ray scattering (GISAXS/T-SAXS) experiments were conducted at P03/MiNaXS beamline at PETRA III / DESY in Hamburg, Germany and are schematically shown in Supplementary Fig. 3c, and Supplementary Fig. 5a, b^28^. The used X-ray energy was 13 keV with a sample-to-detector distance SDD = (3403 ± 1) mm. The two-dimensional scattering patterns were collected using a Pilatus 1 M (Dectris Ltd., (172 × 172) µm^2^ pixel size) detector. The T-SAXS measurements where conducted in a transmission holder using a quartz capillary (Hilgenberg GmbH, diameter = 2 mm, 10 µm wall thickness) at same conditions as the GISAXS experiments. For GISAXS, the X-ray penetration depth was calculated following Dosch et al., and the incident angle was chosen to be *α*_*i*_ = 0.4° to penetrate the full film, see Supplementary Fig. 5c and Supplementary Table 2^29^. Illuminating the sample under conditions where the incident angle is above the critical angle allow the penetration of the full thin film and so yield information over the full thickness^30–33^. The beamline setup includes the UV-LED, a Vis spectroscopy setup to confirm changes in thickness of the thin film during light exposure as well as a camera to observe any macroscopic change. The scattering wavevector $\mathop{q}\limits^{\rightharpoonup}$ is defined as,

1 $$\mathop{q}\limits^{\rightharpoonup} = \left( {\begin{array}{*{20}{c}} {q_x} \\ {q_y} \\ {q_z} \end{array}} \right) = \frac{{2\pi }}{\lambda }\left( {\begin{array}{*{20}{c}} {\cos \alpha _f\cos 2\theta _f - \cos \alpha _i} \\ {\cos \alpha _f\sin 2\theta _f} \\ {\sin \alpha _f + \sin \alpha _i} \end{array}} \right)$$

The sample was kept in a nitrogen-purged chamber with a UV transparent fused silica glass to allow UV irradiation from the UV-LED and X-ray transparent Kapton side-windows to allow the illumination and scattering with X-rays on and from the sample. By conducting the polymerization process in a step-wise fashion, GISAXS and XRR measurements allowed to obtain the two-dimensional scattering pattern that were reduced using the software package DPDAK^34^ and integrated along the Yoneda peak region ($\alpha _f \approx 0.1^\circ$) of the compositions, see Supplementary Table 1. Step-wise means that the UV-irradiation was performed and subsequently the GISAXS and XRR. However, the sample stayed continuously in the sample chamber, and the setup has not been altered during the process. The sample was laterally scanned and exposed to X-rays for 100 ms for each polymerization step to check for homogeneity and to sum (10 patterns) the scattering patterns for higher statistics^35^. The acquisition time of the individual GISAXS pattern was set to 100 ms avoiding any X-radiation induced alterations of the film as demonstrated in Supplementary Fig. 3b. The one-dimensional integrations along *q*_*y*_ were fitted using Pseudo-Voigt function for the Yoneda peak and with a Lorentzian at the peak occurrence from the network formation during light exposure. The fitted q-position was used to calculate the feature in plane length scale, by using the relation $d = \frac{{2\pi }}{q}$. The in-plane correlations in the film before and after polymerization were studied by following the decrease in amplitude of the oscillations from resonant diffuse scattering with increasing *q*_*y*_, see Supplementary Fig. 4^36,37^. The correlation length is calculated by $R_{corr} = \frac{{2\pi }}{{\Delta q_{corr}}}$, where $\Delta q_{corr}$ is the length when the fringes fade away. The correlation length is defined as the measure for the replication of the substrate morphology by the applied thin film. The cut-off length $R_{corr}$ shows the smallest replicated structure sizes within the thin film.

### X-ray reflectivity

The X-ray reflectivity (XRR) measurements were conducted with same parameters as for GISAXS at P03. For the measurements, the initial X-ray beam was attenuated using 0.3 mm aluminum foil to allow only around 40% of the beam intensity to pass to the sample surface. The XRR data was obtained by changing the incidence angle from 0.085° $\to$ 1.8° while integrating one scattering pattern. This procedure was done with two different detector positions to interpolate any detector gaps. The one-dimensional XRR profile was extracted by integrating vertically the scattering patterns at $q_y = 0\,{\mathrm{m}}^{ - 1}$. The resulting profile was fitted with a multilayer model (air, resin, SiO_2_, Si) using PyXRR v. 0.6^38^. All XRR measurements were conducted in consecutive steps between the UV-irradiation.

### Atomic force microscopy

The surface topography was measured for the fully cured thin and thick films. Therefore, a MultiMode (MMAFM-2, Bruker Corporation) AFM with a silicon nitride-based cantilever for soft materials (SCANASYST-AIR-HR, spring constant: 0.4 N/m, resonance frequency: 130 kHz, Bruker Corporation) with a nominal tip radius of 2 nm in tapping mode was used. The topographic measurements were conducted on 3 positions on the sample with a scan size of (10 × 10) µm^2^, to check the surface homogeneity. The root-mean-square roughness was also analyzed from these topographic measurements. For thickness determination, the film was scratched, and the scratch imaged with the AFM. The resulting step was then linearly integrated and leveled to obtain the thickness.

### Dynamic mechanical analysis

The dynamic mechanical analysis (DMA) measurements were conducted to follow the viscoelastic properties of the two different resin formulations. We used a DMA Q800 (TA Instruments) and analyzed the data using the software Universal Analysis 2000 (v. 4.5). For the measurements, undiluted resin formulations were spin-coated on silicon. Due to the differences in viscosity two procedures were followed for depositing the films. In case of LT the film was spin-coated at 800 rpm for 60 s and for HT the film was spin-coated at 1500 rpm for 60 s. Subsequently, the film was kept for 20 min at ambient conditions to level evenly resulting in homogeneous 50–80 µm thick films, which were detached after polymerization from the substrate and cut to (5 × 10) mm^2^ pieces. The DMA measurements were performed in a temperature sweep at a frequency of 1 Hz with a ramp rate of 3 °C/min in a tension stress mode. The temperature ranges were (−60 $\to$ 50) °C and (−50 $\to$ 120) °C for LT and HT, respectively. The phase shift tan δ was evaluated.

## Supplementary information

Supplementary Information

## Data Availability

Raw data were generated at DESY, PETRA III (beamline P03/MiNaXs). Derived data supporting the findings of this study are available from the corresponding author on request.
