# Supplementary material for: Revealing structural evolution occurring from photo-initiated polymer network formation
Source: Commun Chem. 2020 Jul 10;3:88. doi: 10.1038/s42004-020-0335-9 (PMC9814663; doi:10.1038/s42004-020-0335-9)
Supplement: Supplementary file 1 — Supplementary Information [file 42004_2020_335_MOESM1_ESM.pdf]

Supporting Information

Revealing structural evolution occurring from photo-initiated polymer network formation

C. J. Brett[1,2,3*], S. Montani[4,5], M. Schwartzkopf[3], R. A. T. M. van Benthem[6,7], J. F. G. A. Jansen[7], G. Griffini[5], S. V. Roth[3,4*] and M. K. G. Johansson[4*]

[1]KTH Royal Institute of Technology, Department of Engineering Mechanics, Teknikringen 8, 10044 Stockholm, Sweden
[2]KTH Royal Institute of Technology, Wallenberg Wood Science Center, Teknikringen 36, 10044 Stockholm, Sweden
[3]Deutsches Elektronen-Synchrotron, Notkestrasse 85, 22607 Hamburg, Germany
[4]KTH Royal Institute of Technology, Fiber and Polymer Technology, Teknikringen 56, 10044 Stockholm, Sweden
[5]Politecnico di Milano, Department of Chemistry, Materials and Chemical Engineering "Giulio Natta", Piazza Leonardo da Vinci 32, 20133 Milano, Italy
[6]Eindhoven University of Technology, Laboratory of Physical Chemistry (SPC), Groene Loper 5, 5600 MB Eindhoven, the Netherlands
[7]DSM Material Science Center, Urmonderbaan 22, 6167 RD Geleen, the Netherlands
*Corresponding author's e-mail: calvinbr@kth.se, stephan.roth@desy.de, matskg@kth.se

a

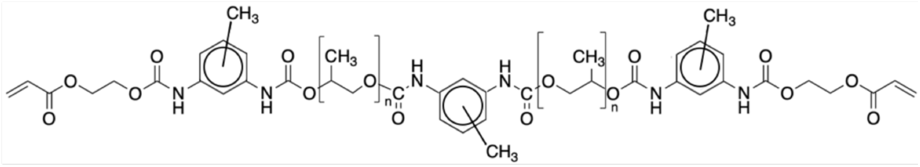

b

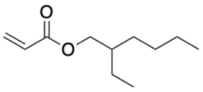

c

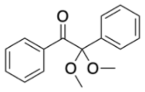

d

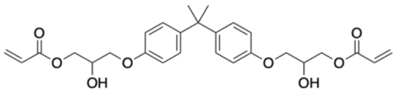

e

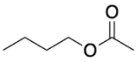

f

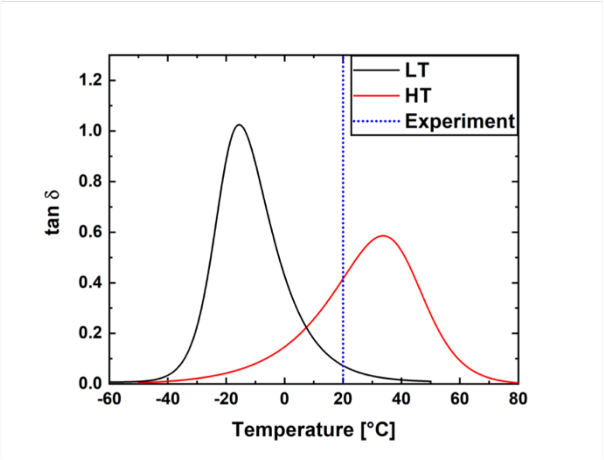

Supplementary Figure 1 Chemical structures for all materials used, the energy dissipation potential tan δ measured by DMA and conversion calculated from FTIR. The chemical structures are shown in a) Polyether urethane diacrylate (oligomer), b) 2-Ethylhexyl acrylate, c) 2,2-Dimethoxy-2-phenylacetophenone (Igacure® 651), d) Bisphenol A diglycidyl ether diacrylate, e) Butyl acetate. f) tan δ measured by DMA and plotted for LT (black) and HT (red) and the corresponding measuring temperature of around 20 °C (blue).

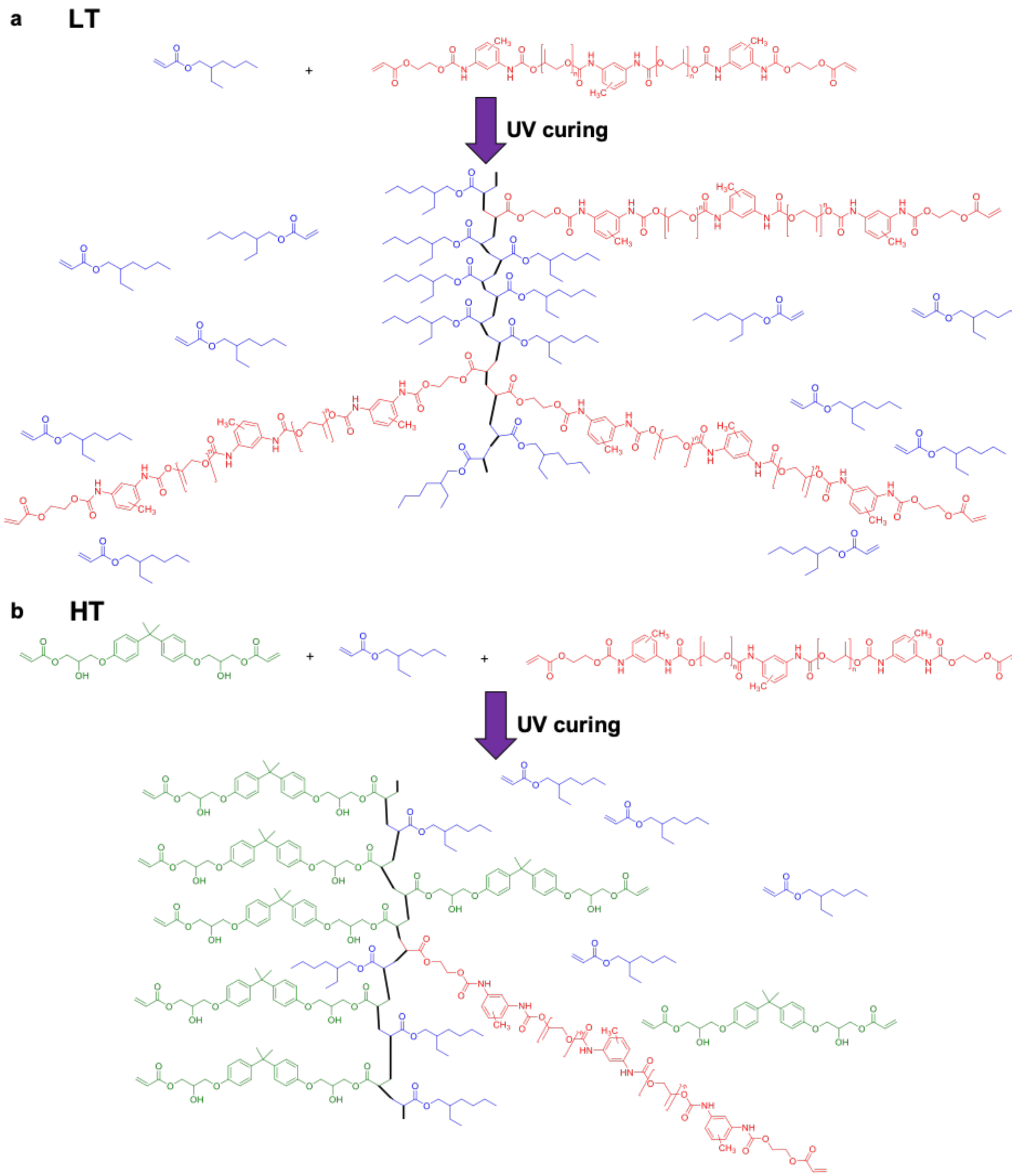

Supplementary Figure 2 Schematic representation of the curing process of the LT (a) and HT (b) formulations. In the initial uncured state (top a) and b)) already a spinodal distribution exists manifest themselves as domains. The initial heterogeneity increases upon curing as cross-linked areas are formed which exhibit not only differences in reactivity due having an acrylate reacted but also due to diffusional limitations and swelling leading to increased domain sizes.[1,2] In case of LT being at RT a mobile network the differences averaging out i.e. it shows a domain feature with the size of the sample. Regarding HT a vitrified network is formed still containing unpolymerized double bonds resulting in a limited increase in domain size.[3]

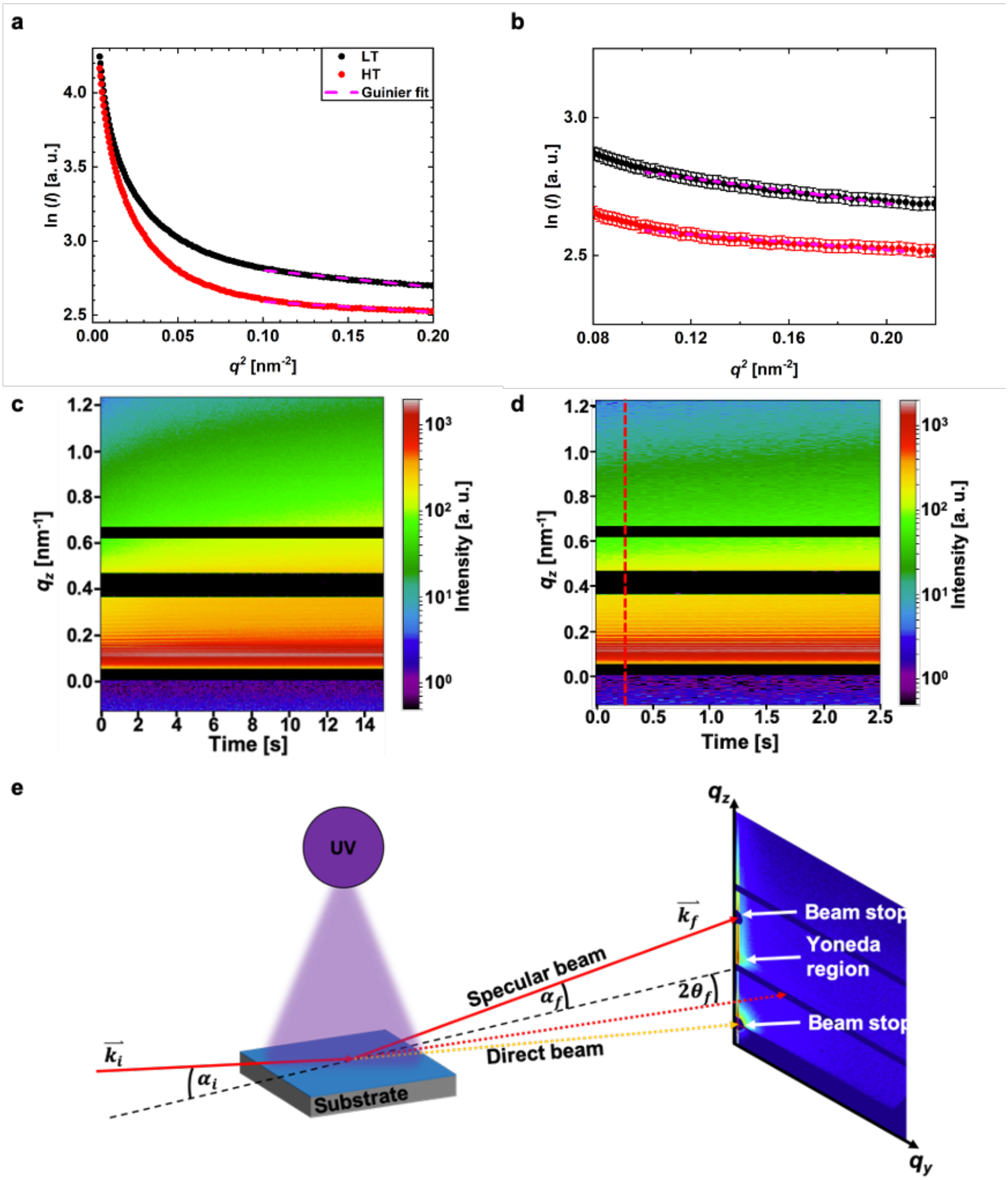

Supplementary Figure 3 Small-angle X-ray scattering analysis, investigation of the X-ray induced structural changes and the schematic GISAXS setup at P03 beamline. a) Guinier analysis of the SAXS data of the pristine initial resins without the photo initiator as well as no solvent. The SAXS data is plotted in ln (*I*) vs. q^2 and fitted in the linear region as $ln\big(I(q)\big) = ln(I_0) - \left(\frac{R_g^2}{3}\right) q^2$ the Guinier radii R_g can be extracted. b) shows the linear region for Guinier fit. c) The integration at $q_y = 0$ nm^{-1} in vertical direction is plotted *versus* the X-ray exposure time at one position of the sample. d) The red-dashed line (0.25 s) shows the start of the induced changes due to the X-ray beam, hence all experiments are conducted below at 0.1 s. e) shows the schematic GISAXS setup *in situ* during the UV-induced photopolymerization experiment. The incident angle is chosen to be $\alpha_i = 0.4°$. The X-ray beam illuminates the substrate surface under a chosen incident angle of $\alpha_i = 0.4°$ and gets scattered according to the distorted wave Born approximation. The scattered intensity is than recorded on a two-dimensional detector. The beam stops shield the direct and the specular beam to prevent saturation and damage to the detector. The region of interest and further evaluated is the Yoneda region.

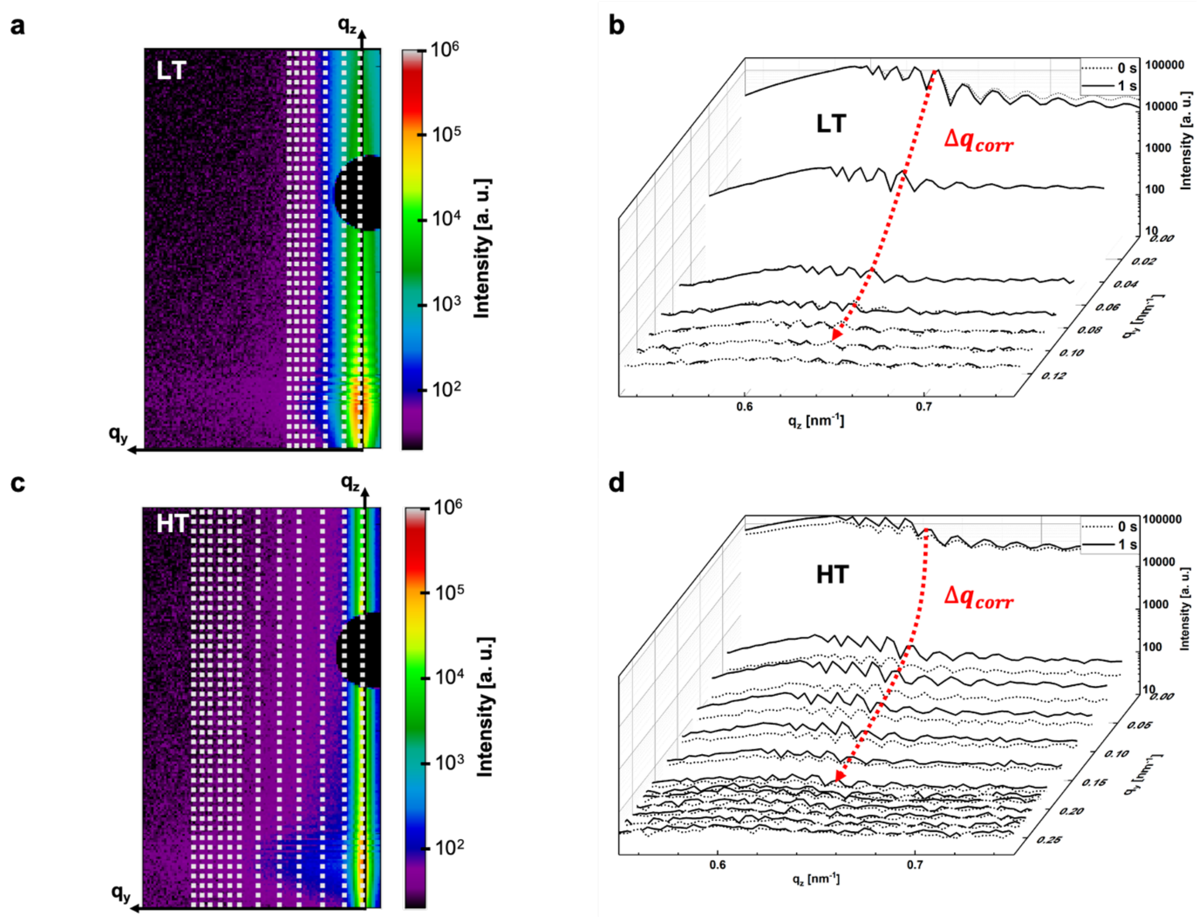

Supplementary Figure 4 In-plane (q_y) correlations extracted from the GISAXS patterns at t = 0 s. a, c) show the scattering pattern for LT and HT correspondingly with vertical integration lines (dashed white). The vertical one-dimensional integration is shown in b) for LT and d) for HT. It is observable that the oscillations in q_z-direction vanish when integrating in higher q_y-values, the dashed red line should help the eye to follow the peaks vanish. The $\Delta q_{corr,LT} =$ (0.12 ± 0.01) nm^{-1} and $\Delta q_{corr,HT} =$ (0.22 ± 0.01) nm^{-1} correspond to a correlation length of $R_{corr,LT} =$ (52.3 ± 4.1) nm and $R_{corr,HT} =$ (28.6 ± 1.2) nm.

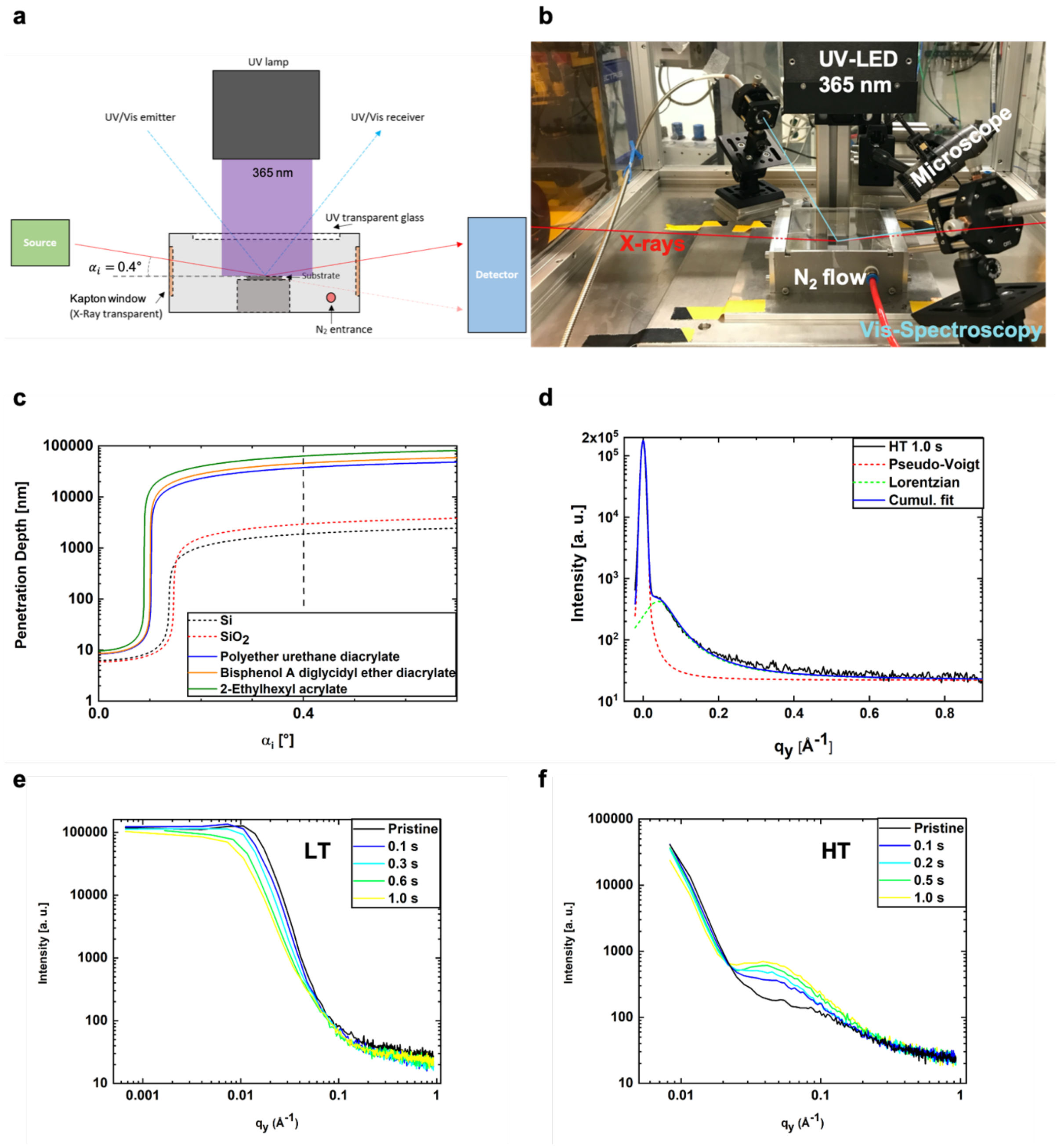

Supplementary Figure 5 UV-polymerization setup at the beamline P03 at PETRA III (Hamburg, Germany). Schematic setup in a) and the picture from the beamline in b). The setup consists of an environmental chamber which is flushed with nitrogen at low pressure to avoid oxygen during polymerization. The chamber has two Kapton windows for illuminating the sample with X-rays and the second window for detecting the X-rays outside the chamber on the detector. The third window on top of the substrate is made of fused silica as this allows the UV-light to pass and polymerize the resin inside the chamber. Additional measurements can be performed during the polymerization, such as Vis-spectroscopy or microscope videos. c) X-ray penetration depth calculated for the materials used in this study. The dashed vertical line shows which incident angle (0.4°) was used, which means the full film is always penetrated. The one-dimensional intensity plot at the Yoneda-region as shown in Figure 2 is exemplarily shown for HT 1 s light exposure. The scattering data is fitted by a sum of pseudo-Voigt – being a linear combination of a Lorentzian and Gaussian curve – for the Yoneda peak profile (red dashed) and a Lorentzian function for the domain peak occurrence (green dashed). The blue line shows the cumulative fit. The 1D integration from the GISAXS patterns in Fig. 2 in q_y-direction are shown in e) and f) for LT and HT respectively.

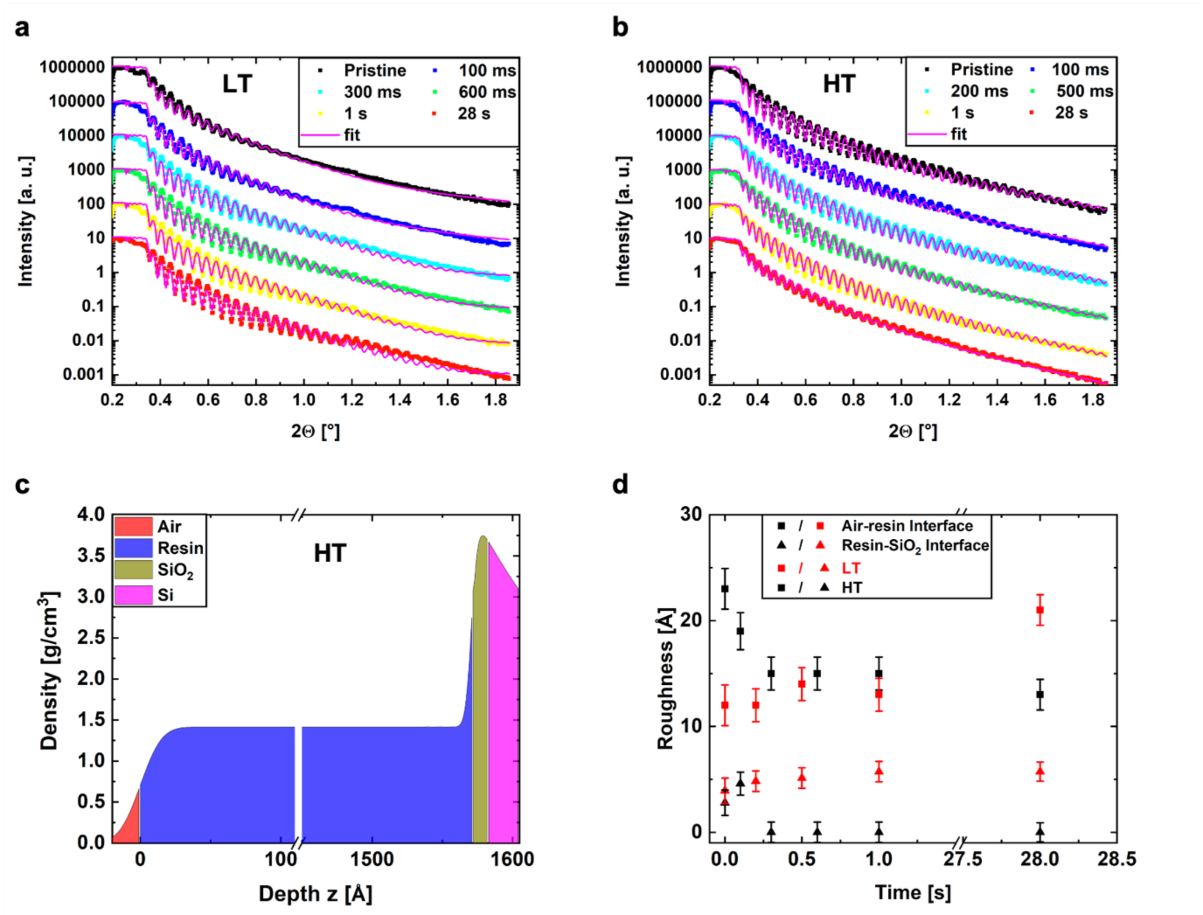

Supplementary Figure 6 XRR plots of the two polymer resins at different steps during the photopolymerization, in a) LT and in b) HT. The fit is represented by a magenta line. All data are fitted using PyXRR v0.6 with a multilayer model as shown in c). The fit for HT in XRR curves resulted in a density plot in c) along the surface normal (z). The fitted multilayer consists of a silicon (Si, magenta) substrate with a native oxide layer on top (SiO_2, beige), subsequently the spin-coated resin layer (blue) and the interface to the air (red). d) The roughness evolution at the air-resin interface (squares) and the resin-SiO_2 interface (triangle) is extracted from the XRR fitting. The polymerization time starts at just spin-coated to fully polymerized at 28 s for LT (red) and HT (black). The error bars result from the fit error and error propagation of the experimental data.

Supplementary Note 1

We fitted a two-layer model including a polymer layer, with a thin silica layer and the silicon substrate. We constrained the polymer layer thickness by means of Kiessig fringes and the substrate. The fitting yielded densities of the polymer layer within a few percentages of the known values. The shown fitting reproduced the curve very well. This yielded the results depicted in Figure 3 and Supplementary Figure 6. It should be noted that different more complex models might fit the data even better; however, many models have been tried and additional layers beneath the polymer layers were giving results of densities <<1 g/cm^3. Being aware of the limitations of our simple model and the performed X-ray measurements, we emphasize that we restrict our interpretation based on the Kiessig fringes and the roughness as well as critical angle. We further want to add that with improving further methods and experiments one could gain more knowledge on these complex resin film layers attached to a substrate.

Supplementary Table 1 Material composition as prepared for LT and HT sample.

	LT [wt%]	HT [wt%]
Polyether urethane diacrylate (oligomer)	71.4	40.8
2-Ethylhexyl acrylate	28.6	18.4
Bisphenol A diglycidyl ether diacrylate	0.0	40.8
**Total**	100	100

Supplementary Table 2 Density ρ, X-ray dispersity δ_n, X-ray absorption β and the critical angle α_c for all materials used in the studied films at 13 keV beam energy.[4] These values were used to calculate the penetration depth in Figure S4.

**Material**	**Formula**	ρ **[g/cm³]**	δ_n **[× 10⁻⁶]**	β **[× 10⁻⁸]**	α_c **[°]**
Silicon	Si	2.33	2.88	2.62	0.137
Silica	SiO_2	2.65	3.27	1.66	0.146
Oligomer	$C_{133}O_{61}N_6H_{233}$	1.20	1.59	0.14	0.102
DGEBA	$C_{21}O_4H_{24}$	1.17	1.54	0.11	0.100
EHA	$C_{11}O_2H_{20}$	0.88	1.20	0.08	0.088

Supplementary Table 3 Exponential fit parameters for FTIR conversion and for the domain sizes deduced by GISAXS. The exponential decay is fitted by $y = y_0 + A_1 \cdot e^{-\frac{x}{t_1}} + A_2 \cdot e^{-\frac{x}{t_2}}$, where x is the time of polymerization and y the conversion in percent for FTIR or the domain size for GISAXS.

	y_0	A_1	t_1	A_2	t_2
LT-Conversion	93.96 ± 1.44	-47.42 ± 2.63	0.16 ± 0.01	-54.09 ± 2.15	2.77 ± 0.29
HT-Conversion	79.29 ± 3.85	-57.93 ± 6.47	0.20 ± 0.06	-27.19 ± 6.73	2.68 ± 1.60
LT-Domains	202.2 ± 16.8	-158.6 ± 17.6	0.59 ± 0.16	N/A	N/A
HT-Domains	16.86 ± 0.22	-3.47 ± 0.35	0.17 ± 0.04	N/A	N/A

Supplementary References

1. Hutchison, J. B. & Anseth, K. S. Off-Lattice Approach to Simulate Radical Chain Polymerizations of Tetrafunctional Monomers. *Macromol. Theory Simulations* **10**, 600–607 (2001).
2. Anseth, K. S., Wang, C. M. & Bowman, C. N. Kinetic evidence of reaction diffusion during the polymerization of multi(meth)acrylate monomers. *Macromolecules* **27**, 650–655 (1994).
3. Krzeminski, M., Molinari, M., Troyon, M. & Coqueret, X. Characterization by Atomic Force Microscopy of the Nanoheterogeneities Produced by the Radiation-Induced Cross-Linking Polymerization of Aromatic Diacrylates. *Macromolecules* **43**, 8121–8127 (2010).
4. Henke, B. L., Gullikson, E. M. & Davis, J. C. X-Ray Interactions: Photoabsorption, Scattering, Transmission, and Reflection at E = 50-30,000 eV, Z = 1-92. *At. Data Nucl. Data Tables* **54**, 181–342 (1993).
